# Farmers' Adoption, Knowledge, and Perceptions of Tick Control Measures on Dairy Farms in Subtropical Areas of Continental Ecuador

**DOI:** 10.1155/2024/5023240

**Published:** 2024-05-24

**Authors:** Valeria Paucar-Quishpe, Ximena Pérez-Otáñez, Richar Rodríguez-Hidalgo, Cecilia Pérez-Escalante, Darío Cepeda-Bastidas, Jorge Grijalva, Sandra Enríquez, Susana Arciniegas-Ortega, Sophie O. Vanwambeke, Lenin Ron-Garrido, Claude Saegerman

**Affiliations:** ^1^ Zoonosis Research Institute (CIZ) Central University of Ecuador Quito 170521Ecuador; ^2^ Research Unit of Epidemiology and Risk Analysis Applied to Veterinary Science (UREAR-ULiège) Fundamental and Applied Research for Animals and Health (FARAH) Center Faculty of Veterinary Medicine University of Liege Liège 4000Belgium; ^3^ Georges Lemaitre Centre for Earth and Climate Research Earth and Life Institute UCLouvain Louvain-la-Neuve 1348Belgium; ^4^ Faculty of Veterinary Medicine and Zootechnics Central University of Ecuador Quito 170521Ecuador; ^5^ Faculty of Agricultural Sciences Central University of Ecuador Quito 170521Ecuador; ^6^ Faculty of Geological, Mining, Petroleum and Environmental Engineering Central University of Ecuador Quito 170521Ecuador

## Abstract

The application of tick control strategies on tropical dairy cattle strongly relies on farmers' uptake, knowledge, and perceptions of the efficacy of control measures. This study aims to identify common and uncommon tick control practices employed by dairy farmers in subtropical areas of Ecuador and associate them with the presence of infestation and acaricide resistance. Data were collected through a cross-sectional survey and participatory meetings. Multiple correspondence analysis was used to explore the association between management variables and the level of tick infestation and resistance. It was determined that the main method of acaricide control is still chemical, mainly using spray baths. Generally, when this form of application is used, acaricides are overdosed, in contrast to the pour-on method with underdosage. Among the measures farmers adopt when chemical treatment has failed is to use overdoses of products, mix different acaricides, and use focused treatments (wipe cloth) with irritant substances. The absence of a high level of infestation was related to acaricide dips every 3–4 weeks and the use of intensive grazing. On the other hand, the high infestation was related to the use of organophosphates, wipe cloth application, and the report of tick-borne diseases (TBDs). A small group of farmers have good knowledge and seek alternatives to chemical control, experimenting with biological controls, herbal extracts, manual tick removal, and paddock control. Additionally, farmers reported the presence of TBDs (47%) and the presence of animals poisoned by acaricides (6%), which died in 75% of those cases. Farmers frequently mentioned that tick infestation induces milk drop production and weight loss and is associated with the presence of TBDs. This information is crucial to improve tick control management in Ecuador, particularly through implementing practices that mitigate resistance to acaricides and ensure long-term solutions that help maintain the efficacy of tick control treatments.

## 1. Introduction

The cattle tick *Rhipicephalus microplus* is a major cause of concern for cattle breeding in the tropical and subtropical areas of the world [1]. Although there are different methods of tick control in cattle, such as immunological control through vaccines, selection of tick-resistant cattle breeds, grazing management, manual removal of ticks, biological control, and the use of ethno-veterinary practices (herbal extracts), chemical control remains the primary method for tick control [2, 3].

In Ecuador, *R. microplus* is the main cattle tick [4, 5, 6, 7], and several acaricides are available for its control, with different active compounds, modes of action (Table 1), and application forms. A wide range of active compounds exerts their action at multiple points in the nervous system of ticks [17]. The first acaricides to be introduced in Ecuador were organophosphates, amides, and synthetic pyrethroids. By the end of the 1990s, macrocyclic lactones and phenylpyrazolones were already available on the market [18], and in the 2000s, the acaricide fluazuron (benzoylphenyl urea), belonging to a new category called insect growth regulators, was introduced to the market in Ecuador [18]. Unlike the acaricides mentioned above, fluazuron inhibits the molting process of tick larvae to nymphs and nymphs to adults [19, 20]. Although the choice of acaricide treatment to be used on the farm will depend mainly on the farmers, they usually receive advice from public and private veterinarians and commercial representatives of products. In addition to chemical control, the Gavac vaccine entered the market in 2022. However, farmers still lack confidence in its effectiveness as a control method due to the investment involved in its application, its unknown efficacy, and the fact that it must be used with a chemical treatment [21].

Although the chemical method was initially considered the primary strategy to control tick infestations, its inadequate management has led to the emergence of acaricide resistance and incurred additional costs associated with reported cases of resistance in the country [6, 7, 22, 23]. Furthermore, there is a concern about environmental contamination and the potential presence of acaricide residues in dairy and meat products, despite no reported cases in Ecuador, as their existence is known [24, 25, 26].

These problems highlight the need for refining the practices and open the window for alternative approaches to control tick infestations. Integrated tick management (ITM) consists of a combination of tools and strategies to manage tick infestations while maintaining adequate levels of animal production [27, 28, 29]. Implementing these strategies requires the appropriate acaricide management to ensure effective and sustainable control practices [30]. The ITM's success depends on individual actions, specifically the acceptance and application of recommendations provided by technicians, as well as on government policies that implement extension programs in the livestock industry, which are currently limited or nonexistent for small farmers [31, 32, 33].

Jack et al. [30] mentioned that several fundamental factors are involved in the process of implementing new control methods, such as farmer characteristics (knowledge, motivations, economics), local support organization (resources, priorities), and the interventions employed (training, leadership). Likewise, this study was built upon two previous studies that aimed to understand livestock practices and examine the level of tick infestation and the development of acaricide resistance in two subtropical areas in Ecuador with the dairy industry. Considering the challenges faced by the livestock industry in these study areas, this study tries to integrate various aspects, including perceptions, knowledge, infestation levels, and acaricide resistance found in these areas [7, 23]. Thus, the objective of this study was to qualitatively evaluate the perceptions, knowledge, and common tick control practices used by dairy farmers in subtropical areas of continental Ecuador and to associate them with the presence of infestation and acaricide resistance.

## 2. Materials and Methods

### 2.1. Participant Selection

Two main methods were employed in this study (Figure S1). First, a cross-sectional survey was carried out in two subtropical dairy production areas of Ecuador: Area 1, located in the Northwest of Pichincha Province in the Western Andean foothills, and Area 2, situated in the Quijos river valley in the Eastern Andean foothills. The participants were selected by snowball sampling [34] irrespective of their age, sex, and educational background. The only requirement to participate was to be the most knowledgeable person on the farm. Special emphasis was placed on including farmers from small and medium-sized cattle ranches. Verbal informed consent was obtained from all participants.

The second part of the involved study was conducted in each study area using participatory methodology [35]. The attendees of these meetings were cattle ranchers of any age, sex, and education level. Participants were identified and invited either by phone or in person with the assistance of local government officials. Transportation was provided for the workshops to facilitate access.

### 2.2. Data Collection

#### 2.2.1. Cross-Sectional Survey

The survey was part of the project “Socio-eco-epidemiology of ticks, tick-borne parasites, acaricide resistance and residual effects of acaricides in Ecuadorian tropical livestock: environmental, animal and public health impacts” [7]. In total, 138 farmers were interviewed, 71 from the Northwest of Pichincha province and 67 from the Quijos River valley in Napo province. This face-to-face survey contained questions on herd management, livestock diseases, ticks, and acaricide-related information such as perceptions, knowledge, and acaricide control methods. Commercial acaricides were grouped according to their active ingredient into amides, pyrethroids, macrocyclic lactones, organophosphates, phenylpyrazolones (fipronil), benzoylphenyl ureas (fluazuron), and combined products. In addition, information about the doses used, perceived efficacy, application method, number and kind of animals treated, acaricide rotation, and prices per product were recorded. The rotation of acaricides was considered incorrect if either different acaricide brands were within the same acaricide group or if the farmer was not sure of the brand name of the acaricide used previously. The efficacy evaluation was expressed in percentages according to the farmer's perception (1–100). Price information was confirmed by an additional interview conducted in the livestock warehouses in the study areas [23]. Local veterinarians were asked to mention the main diseases or events affecting animals in the area, and this list was used in the farmers' survey to assess general prevalence and mortality.

Farmers were asked to visually identify the species of cattle ticks they encountered based on illustrations of common species found in the study areas [36, 37, 38]: *R. microplus*, *Amblyomma cajennense* and *Ixodes boliviensis*. In addition, all farmers were asked to indicate the season (dry season, rainy season, all over year) and the months during which tick infestation increases. Farmers' knowledge was evaluated with six elements: the biology of ticks, breed predisposition, tick-borne diseases (TBDs), knowledge of economic losses caused by ticks, and correct acaricide treatment. Knowledge of biology consisted of the correct identification of the tick species present on the farm and its life cycle (presence of larvae in paddocks). Morphological identification was carried out on tick samples [7]. Knowledge of TBDs was assessed as correct if the farmer mentioned anaplasmosis, babesiosis, or tick fever (the colloquial name for TBDs).

#### 2.2.2. Participatory Meeting

**Figure 1:**
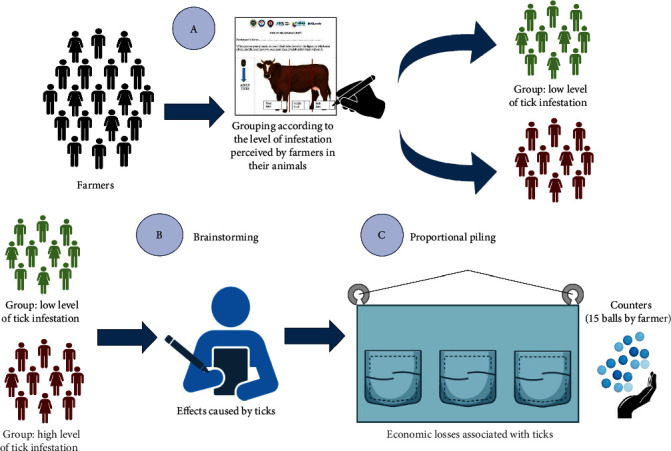
Methodologies used in the participatory meeting.

Participatory methods are methods to collect data in participatory epidemiology, engaging communities in the surveillance, control, and prevention of animal diseases [35, 39]. Those observations play a crucial role during early detection and community response to diseases' effects. Perceptions of the effects caused by ticks and the economic losses they cause were evaluated in this part according to the opinion of the farmers. The methodologies used for this involved proportional piling and brainstorming (Figure 1).

The second part of the study was conducted in April 2022. Forty farmers participated, 13 from the Northwest of Pichincha and 27 from the Quijos River valley. We used proportional piling, which allows to collect the results numerically [35]. First, farmers were classified based on their perception of the level of tick infestation present in their animals. At the beginning of the participatory meetings, the participants were grouped in function of the response given to an illustration of a laterally viewed cow divided into three zones (Figure S2). Farmers indicated which parts of the animals were infested and considered one-third as infested when there were 20 or more ingrown ticks. The possible options were to have one-third to three-thirds infested. It was considered a low level of infestation if it was one-third infested, a medium level of infestation two-thirds, and a high level of infestation three-thirds infested [7]. Participants in the last two groups were grouped in the high level of tick infestation category.

Second, for brainstorming, each participant had four cards to write down their ideas about the effects of ticks. Only one idea was recorded per card. Using all cards was optional, and additional cards were provided upon request. An assistant helped to assist illiterate and older participants. Finally, the economic losses resulting from decreasing milk production, weight loss, and the devaluation of hides were weighted by proportional piling. During the exercise, meeting attendees engaged in a proportional piling activity, using balls as counters. A cloth with pockets was used for the proportional measurement to prevent the first participant's response from influencing subsequent responses. Fifteen counters were employed to weight these apparent economic losses.

### 2.3. Tick Infestation and Acaricide Resistance

The levels of infestation and the presence of acaricide resistance were determined in previous published studies [7, 23]. The level of infestation at the farm level (low or high) was determined according to the tick load observed per animal. Resistance testing was performed on three acaricides (i.e., amitraz, ivermectin, and alpha-cypermethrin) using the larval package test. The farms were classified as with and without acaricide resistance.

In the study, the variables considered to estimate the tick control practices and the economic losses are presented in Table 2.

### 2.4. Data Analysis

All the information collected in the cross-sectional survey and participatory meetings was entered into a Microsoft Excel® database. In order to preserve the anonymity of the study participants, the surveys were coded with numbers (participating farmer numbers) and letters (study area). The average price per milliliter (ml) or gram (g) of the active component of the acaricide was obtained by dividing the price of the commercial presentation by the number of ml or g. All data obtained for the different commercial presentations were averaged, and a price per ml or g of active ingredient was determined.

The prescribed dose, route of application, and composition of each commercial brand of acaricide were obtained from the package inserts. For injectable acaricides, the prescribed dose was 1 ml per 50 kg of body weight, and for pour-on acaricides, 1 ml per 10 kg of body weight. In the case of acaricides applied in spray baths, the dose was expressed in milligrams or grams of acaricide dissolved per liter of water, and 1 l of solution covers 100 kg of body weight. To calculate the cost per acaricide treatment, an adult animal weighing 400 kg was considered. To determine if there was a significant difference between the dose and the prescribed dose, a *t*-test was used. The prescribed dose was used as the real value of the mean. Statistical significance was set at 0.05. Statistical analyses were performed using the statistical package stats in R (R Core Team) [40], version 4.2.0. The level of agreement between the working groups was evaluated using Kendall's coefficient of concordance (*W*). The agreement was termed “weak agreement” if *W* values were less than 0.26, “moderate agreement” if they were between 0.26 and 0.38, and “strong agreement” if *W* values were greater than 0.38 [41]. Mann–Whitney test was used to analyze differences between infestation level and study areas.

Multiple correspondence analysis (MCA) was used to summarize associations between the risk practices in tick control, knowledge regarding ticks and TBDs, level of tick infestation, and acaricide resistance. The MCA is a statistical method to analyze patterns in the relationships between a set of qualitative variables, and its interpretation is based on proximities between points in a low-dimensional map [42]. The hierarchical classification on the principal components (hierarchical clustering on principal components (HCPC)) of the MCA was used for the clustering process. We used the FactoMineR package [43] in R to perform MCA and HCPC analyses. The functions fviz_mca_var and fviz_cluster (factoextra package) were used to visualize the results [44]. This study used 20 variables (Table 3) to establish the relationship and grouping farmers according to control practices and perceptions. Covariates with little or no variability were discarded from the analysis. Twelve farms were discarded from the MCA because acaricide resistance tests could not be performed there. The knowledge was judged as good, fair, or poor based on the number of correct answers. Poor knowledge meant zero to <35% of correct answers, fair knowledge was 35%–65% of correct answers, and good knowledge was >65% or all correct answers [45]. The reported efficacy of the acaricide treatment was grouped into three categories: low, medium, and high. Medium efficacy grouped reports of efficacy from 51% to 80%. Alternative control included acaricide control with entomopathogenic fungi, medicinal plants, or paddock control (equalization cuts).

## 3. Results

### 3.1. Visual Identification and Cattle Tick Seasonality

*R. microplus* was the main species recognized in the study areas. *R. microplus* was visually identified by 94% of the farmers in the Quijos River valley and 93% in Northwestern Pichincha as the species that attacks their animals. Ticks of *A. cajennense* were recognized by 29% and 24% of farmers in the Quijos River valley and Northwest Pichincha, respectively. Finally, *I. boliviensis* was recognized by 10% of farmers in the Quijos River valley and 12% of farmers in Northwestern Pichincha.

**Figure 2:**
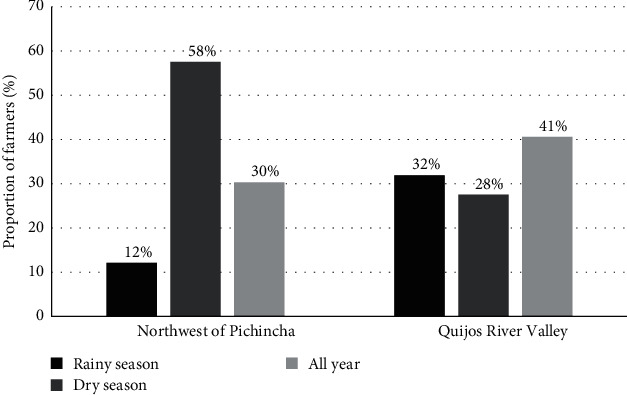
Perceptions of seasonal abundance of ticks (*N* = 138 farmers considered).

Figure 2 illustrates the perception of the seasons with the highest tick infestation. Farmers in the Quijos River valley reported experiencing infestations throughout the year. Most farmers in Northwestern Pichincha reported an upswing in infestation during the dry season, from July to September.

### 3.2. Farmers' Knowledge

**Figure 3:**
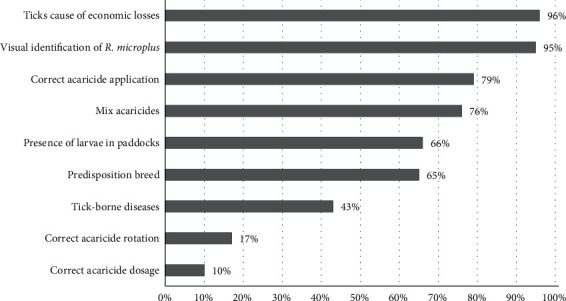
Farmers' knowledge about ticks and tick-borne diseases.

Knowledge was evaluated according to the items shown in Figure 3. Seventy percent of participants had fair knowledge, and 27% had good knowledge. Three percent of the participants had poor knowledge. The farmers with “good knowledge” demonstrate excellent knowledge of the biology of ticks, the diseases they transmit, the economic losses they cause, and the breed predisposition. However, their understanding of the correct acaricide management is neither exceptionally excellent nor bad; it falls within an intermediate range. The “fair knowledge” group differs from the farmers with good knowledge because of a lack of understanding of TBDs and the correct use of acaricides.

The results indicate that a significant number of farmers require further understanding of the appropriate acaricide dosage and rotation, with only 10% and 17% demonstrating correct management, respectively.

### 3.3. Chemical Acaricide Control and Perception of Its Efficacy

**Figure 4:**
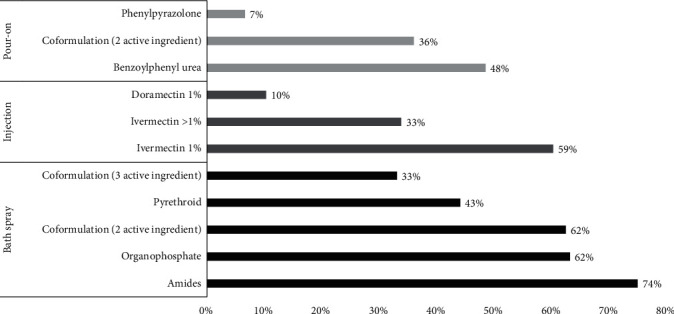
Acaricides used by farmers (*N* = 136) in the study areas, expressed in percent.

Of the 138 farms surveyed, 99% reported using chemical acaricide control. The most common form of application was spraying (95%, 131/138). Acaricides used as sprays can be mono-formulated with organophosphates, amides, and pyrethroids or coformulated with two (organophosphates and pyrethroids) or three active ingredients (organophosphates and pyrethroids and phenylpyrazolones) (Figure 4). Spraying baths are carried out using a 20-l knapsack sprayer. The most common equipment used for acaricide measurement were syringes (74%) and ungraduated acaricide bottle tops (20%). Only 6% of the participants stated that they did not measure the quantity used with any instrument. The most common water sources for mixing acaricides were tap water (11%) and water collected from natural reservoirs (89%) such as rivers, springs, wells, or drainage ditches. Injectable acaricides (Figure 4) were used by 86% of the respondents with macrocyclic lactones such as doramectin at 1% (10%) and ivermectin (90%), at concentrations ranging from 1% to 4%. Ivermectin 1% is the most commonly used (59% of the cases), followed by ivermectin 3.15% (25%) and ivermectin 4% (9%). Additionally, 52% of the respondents employed pour-on acaricides, a relatively new control method in our study areas. Pour-on acaricides can be composed of phenylpyrazolones or benzoylphenyl ureas or coformulated with two active compounds, such as the combination benzoylphenyl ureas (fluazuron) with phenylpyrazolones (fipronil), macrocyclic lactones (abamectin) or pyrethroids (flumethrin). In most farms, the weight of animals is unknown, with only 5% of the respondents utilizing weigh tape to determine the appropriate dose of acaricides. The rest of the farmers calculate the weight of the animals by visual assessment.

Only 17% of farms rotated acaricides correctly, 63% rotated incorrectly, and 19% cannot remember the previous acaricide used. Acaricide treatments in bath sprays and pour-on are generally applied to all animals on the farm. Farmers applied 1% macrocyclic lactones to cattle in production and calves and preferred ivermectin concentrations of 3.15% or 4% for dry cattle, bulls, and heifers. Only 4% of the respondents said they applied an acaricide treatment only to affected animals.

There were many acaricides with the same chemical composition but with different manufacturers and trade names. Approximately 67 different trade names are employed for the six active ingredients available on the market in the study areas. Ten trade names of amides, 13 of cypermethrin, eight for organophosphates, 26 of macrocyclic lactones, five of phenylpyrazolones, and five of benzoylphenyl ureas were mentioned.

The reported efficacy of the chemical acaricides is summarized in Table 4. Respondents reported an efficacy of 59% for amides and pyrethroids; the acaricides were considered the least effective in acaricide control. Pour-on acaricides and macrocyclic lactones (3.15% and 4% concentration) were considered more effective; the farmers perceived the efficacy to be greater than 82%. Unfortunately, farmers do not respect the doses prescribed by the manufacturers (*p*-value < 0.05). Acaricides applied in spray baths were generally overdosed and cost much less than pour-on acaricides, which were generally used in underdoses. Acaricides applied by spraying are more economical compared to injectable and pour-on treatments.

### 3.4. Alternative Strategies Used by Farmers When Chemical Control Fails

Among the alternatives to chemical control, 36% of the farmers used equalization cuts in paddocks, and 5% of the respondents used baths with entomopathogenic fungi, herbal extracts (neem or garlic), or a mixture of sulfur and quicklime. Supplementation with sulfur in mineral mixtures or feedstuffs was also used as an alternative control method, and only two farms used these alternatives as the only control method. These alternative control methods are mostly used in the Northwest of Pichincha in addition to chemical control. The survey also reported that 11% of farmers use lemon, citric acid, or vinegar to acidify the bath solution because they perceived that it increases the efficacy of the acaricide. Thirty-three percent of respondents reported manually removing ticks from the animals. This is a laborious technique that farmers generally do not set aside a specific time for but do while milking.

The unusual forms of application were observed and 12% of respondents who use pour-on reported dissolving them in water and applying them with a spray pump. Eleven percent of farmers reported dissolving an overdose of the acaricide (up to five times the recommended dose) in water, cooking oil, or engine oil and applying it with a wipe cloth to the most affected areas. In addition, 25% of the farmers mixed different acaricides when preparing the solution for spraying. Generally, when using these techniques, the acaricides used are organophosphates.

### 3.5. Chemical Handling Safety

When applying the baths spray, none of the surveyed farmers used all individual protective equipment (coveralls, boots, masks, gloves, and goggles). Instead, they opted for various types of protective equipment, with boots (72%), followed by masks (45%), coveralls (24%), gloves (26%), and goggles (6%). Additionally, 22% of respondents mentioned not using any protective equipment. In addition, 48% of respondents reported taking a shower and changing clothes after spraying. Thirteen percent washed their hands and changed clothes. Seventeen percent only washed their hands, and 22% continued their work in the field without any posterior clean. Sixteen percent of farmers reported having at least one of the following signs: dizziness, vomiting, reddening of the skin, tearing, red eyes, and difficulty breathing after spraying animals.

### 3.6. Risk Management Practices

**Figure 5:**
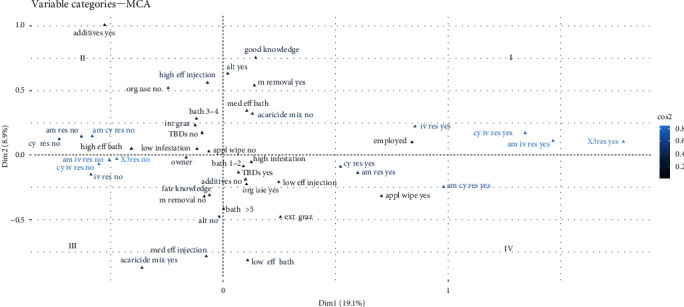
Multiple correspondence analysis map of risk and perceptions associated with the presence of infestation and acaricide resistance. I, first quadrant; II, second quadrant; III, third quadrant; IV, fourth quadrant; Dim1, first dimension; Dim2, second dimension; cos2, squared cosine. See Table 3 for the meaning of variable codes. To interpret the graph, the light blue-colored categories are considered to have the strongest contributions, whereas the dark blue ones are the least. Additionally, points located close together within the same quadrant and aligned in a similar direction from the centroid indicate potential associations.

The first two dimensions of 23 provided by the MCA were retained as they accounted for 19.1% and 8.9%, respectively (Figure 5).

The first dimension can be interpreted as a gradient of “acaricide resistance” since the variables that contributed most were the variables of resistance and (multi)resistance to acaricides. The second dimension can be interpreted as a gradient of “acaricide control practices” since the variables that contributed most were level of knowledge, acaricide control practices (mixing acaricides and alternative control practices), and perception of the efficacy of acaricide control. In Figure 5, the first quadrant (I) shows the relationship between the presence of acaricide resistance and high infestation with good acaricide control practices. The second quadrant (II) shows the relationship between the absence of acaricide resistance and high infestation with good acaricide control practices. The third quadrant (III) shows the relationship between the absence of acaricide resistance and high tick infestation with poor acaricide control practices. The fourth quadrant (IV) shows the relationship between acaricide resistance and high infestation with poor acaricide control practices.

The results of the MCA analysis revealed several associations within the study. Acaricide resistance was associated with the use of a wipe cloth for acaricide application and if the employee was responsible for preparing and applying the acaricide treatments. Conversely, the absence of resistance was related to if the owner was responsible for preparing and applying the acaricide treatments and also the perception of high efficacy in acaricide treatments used in spray baths. Additionally, a high level of tick infestation was associated with factors such as the frequency of acaricide treatment, very frequent (1 or 2 weeks) or not regular (5 or more weeks) bath spray, the use of organophosphates, fair knowledge, low perception of injectable acaricide efficacy, reported TBD cases on the farm, and extensive grazing. A low tick infestation was related to not using a wipe cloth to apply the acaricide, using bath spray every 3–4 weeks, intensive grazing, the nonuse of organophosphates, and the absence of TBDs on the farm. The low perception of spray baths' efficacy was associated with the mixture of acaricides and the relative medium efficacy of the injectable treatment.

Furthermore, participants with good knowledge demonstrated a higher likelihood of adopting alternative methods to chemical control, such as equalization cuts, entomopathogenic fungal dips, herbal extracts, sulfur, or manual tick removal. In contrast, those with fair and poor knowledge displayed a lower tendency to implement alternative control methods.

**Figure 6:**
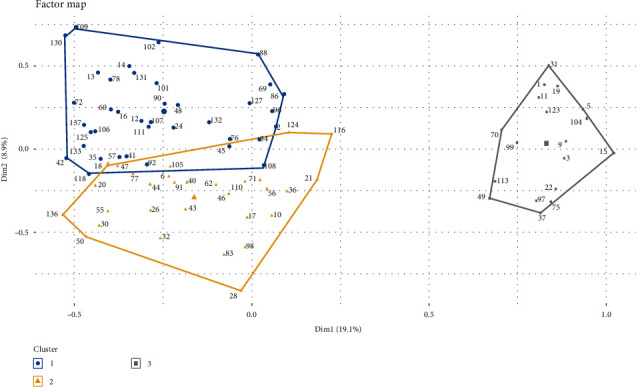
Graphical representation of farmers identifying three clusters (groups). Dim1, first dimension; Dim2, second dimension. Group 1 in blue color; Group 2 in yellow color; Group 3 in gray color.

Three farm clusters were identified through the MCA and subsequent hierarchical classification (Figure 6 and Table 5). The first cluster included 35% of farmers, and it was characterized as follows: 29% of farms with no resistance, 42% with mono-resistance, and 29% with multiresistance to two acaricides. This cluster reported a high infestation in 39% of cases. Generally, acaricides were not applied with a wipe cloth in this group, and the use of organophosphates (47%) was not common. Furthermore, only a few farms mixed different acaricides (13%). Notably, most farmers with good knowledge (12/25) and farms implementing alternative methods 32/55 of acaricide control or adding additives (8/10) to the acaricide solution were concentrated in this first cluster. The second cluster encompassed 44% of farmers and was characterized by farms with varying resistance profiles: 23% without resistance, 37% with mono-acaricide resistance, and 47% with multiresistance to two acaricides. In this cluster, the high infestation was prevalent in 57% of cases. Most farms in this group used organophosphates (93%) and mixed acaricides (53%). In addition, 10 of the 55 farms using alternative control methods were classified under Group 2. The third cluster included 21% of farmers and was distinguished by farms showing multiresistance to three acaricides, with high infestation reported in 50% of cases. Generally, farms in this group did not mix different acaricides but did use organophosphates (78%). Thirteen of the 55 farms using alternative control methods were classified within this group.

Farms that applied acaricides using a wipe cloth were distributed across Groups 2 and 3. Additionally, in these groups, the frequency of acaricide treatment by spray bath was generally every 2 weeks or less. Cases of tick fever were reported, and extensive grazing was employed in around 50% of the cases. In contrast, the frequency of bath spray occurred every 3 or 4 weeks, and a few farms experienced TBDs (34%). For Groups 1 and 2, the person in charge of preparing the acaricide solution and applying the treatment was typically the owner. In Group 3, the owner's participation was present in only 50% of the cases. Regarding the perception of efficacy, most farmers in all three groups rated the efficacy of the acaricide bath as “medium efficacy,” while the injectable treatment was rated as “high efficacy” by farmers in Group 1.

### 3.7. Most Frequent Cattle Diseases Cited by Cattle Farmers

**Figure 7:**
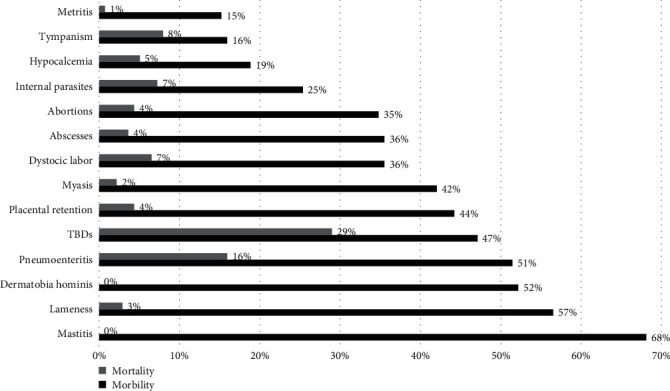
Percentage of farmers (*N* = 138) who cited the disease (morbidity and mortality). TBDs, tick-borne diseases.

According to the farmers' reports in the study areas, health problems among livestock are primarily dominated by mastitis (68%) and lameness (56%). Myasis due to *Dermatobia hominis* (Linnaeus) was reported in 52% of the farms surveyed. TBDs were reported by 47% of farmers and they have the highest mortality on the farms studied. According to reports from farmers on 40 of 138 farms, there was mortality due to TBDs, accounting for 29% of the total deaths. In addition to the diseases described in Figure 7, the study areas also experienced incidents of animals falling down ravines (33%) and animals getting intoxicated by acaricides (6%), leading to mortality of 21% and 4%, respectively.

### 3.8. Effect of Tick Infestation

The effects of infestation were investigated through both the survey and the participatory meetings. Among the farmers surveyed, 94% reported that cows in production were most affected by ticks, followed by calves (43%), dry cows (40%), and bulls (34%). Moreover, 90% of the surveyed indicated that ticks were most abundant on the front third of the animal, with 75% reporting their abundance on the back third and 34% on the middle third.

**Figure 8:**
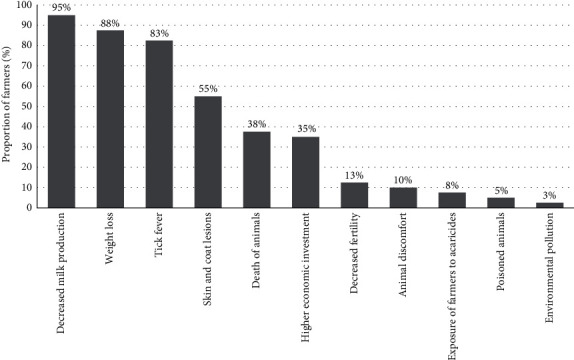
Perceptions of the effects caused by tick infestation according to farmers using participatory meetings, in decreasing order (*N* = 40 farmers considered).

Meeting participants listed several effects of tick infestation (Figure 8), the most common of which were decreased milk production (95%), weight loss (88%), and tick fever (83%). Decreased fertility (13%), animal discomfort (10%), exposure of farmers to acaricides (8%), poisoned animals (5%), and environmental contamination (3%) was reported by a minority of participants. Animal death (38%) and skin and coat lesions (55%), including hair loss, scaling, and attack by other ectoparasites, were also mentioned. Higher economic investment (35%) encompasses the purchase of more expensive acaricides and treatment costs when animals become ill with tick fever.

The matrix scoring results of disease signs are shown in Table 6. The highest weighting was for economic losses caused by the decrease in milk production in farms with or without high infestation in both study zones. Economic losses caused by body weight loss obtained a higher weighting in the Northwest of the Pichincha zone. In contrast, the economic losses due to the decrease in the price of bad hides had a higher weighting in the Quijos River valley zone.

The results of Kendall's *W* are shown in Table 6. The level of agreement was weak for the high infestation group in the Quijos River valley (*W* = 0.16). On the other hand, the groups of low infestation in the two study zones (*W* = 0.53–0.84) and high infestation in the Northwest of Pichincha zone (*W* = 0.74) strongly agreed. In the Northwest Pichincha, the economic losses in milk, weight, and hide of the low infestation group were largely similar to the findings of the high infestation group. The same occurred in the Quijos River valley area, between the high and low infestation groups for meat and hide losses. Only for the low infestation group in Quijos River valley, there was a significant difference between the opinions of these groups on economic losses caused by decreased milk production.

## 4. Discussion

### 4.1. Tick Control

The present study documents common and uncommon tick control practices in cattle in subtropical areas of Ecuador. It was determined that the main method for tick control is chemical control in bath spraying, which smallholder farmers consider a convenient and economical form of control [46, 47, 48]. The costs of acaricides applied in bath sprayings usually do not exceed USD 0.55 per animal per treatment, and farmers reported efficacy ranging from 59% to 71%. Although the treatment by bath spraying was assessed to be moderate, it is important to note that this evaluation does not necessarily indicate correct use. In fact, observations revealed that these acaricides are commonly overdosed. Treatments applied in pour-on form are the most expensive, costing up to USD 3.62 per animal per treatment. By contrast, these acaricides usually are underdosed, likely in relation to their high cost [49], which may contribute to resistance in the long term, previously described in other Latin American countries since 2007 for fipronil [50] and 2014 for fluazuron [1]. Although the price difference of acaricides can influence the farmer's decision on which product to buy and use since it is a more tangible cost, especially for the small farmer, relying solely on price differences does not provide a comprehensive assessment of a product's profitability over time. To accurately determine profitability, one must also evaluate the associated costs of labor, infrastructure, and treatment frequency in conjunction with the product's value. In addition, acaricides that are applied by pour-on cannot be administered to lactating dairy cattle, so they cannot be used as a unique control measure on dairy farms. A comprehensive approach that combines various control methods is necessary to ensure effective tick management.

Coformulated acaricides stands out as they are used by 83.33% of the surveyed farmers in spray baths or pour-on. While a reduction in the concentration of active ingredients in coformulation compared to their corresponding mono-formulations is justifiable under ideal conditions, their effectiveness may diminish in cases where resistance to one of the active ingredients has already developed. “In such scenarios, exposure to suboptimal concentrations could accelerate the emergence of resistance against an otherwise effective molecule present in the coformulation due to inadequate exposure doses” [51]. Additionally, it was found that 25% of farmers make their own coformulated acaricides by blending different commercial acaricides, a practice reported in other studies, where farmers try to maximize and prolong the acaricidal effect [47]. While using coformulated acaricides purchased or modified by farmers may be perceived as an innovative and potentially more effective approach, it poses several concerns. It increases the costs associated with acaricide treatment and increases the risk of acaricide resistance [23, 46]. Moreover, the use of these mixtures raises the risk of poisoning in humans and animals (6% of animals getting intoxicated by acaricides in this study), as the combinations can significantly exceed the permitted dosage levels, particularly when the same active ingredient is used.

It was observed that farmers with “good knowledge,” in addition to chemical control, have implemented other methods for acaricide control, such as manual tick removal, the addition of additives to regulate the pH of the water, use of entomopathogenic fungi, herbal extracts or grazing management. Although manual tick removal is only used by 33% of the surveyed farmers, as it is considered a tedious technique, it is known that if it is performed twice a week during milking, it reduces 21% of the parasite population [52, 53, 54]. Both the use of entomopathogenic fungi and herbal extracts are practiced at a very low rate by farmers in the study areas (5%). Although the use of entomopathogenic fungi has been a practice used in other countries for several years, in Ecuador, this alternative is relatively new and has been gaining importance since 2019 [55, 56, 57, 58], so we associate its low use to the lack of knowledge of the technique and/or its unknown effectiveness in the field. The same occurs with herbal extracts such as neem or garlic, used in a few farms in the study areas as an alternative to chemical control. Neem oil and garlic extracts have potential as acaricides and insect repellents. Neem plant contains azadirachtin, a chitin inhibitor, and garlic contains about 94% of volatile sulfur compounds acting as a repellent [3, 59, 60]. Following the line of acaricide control with sulfur, some farms have implemented control through baths with a mixture of sulfur and quicklime and/or the addition of sulfur to animal's diet, which, according to studies conducted in the study areas, seems to be a viable option [61]. In addition, its efficacy has already been studied in other mites, such as spider mites [62].

Another form of control alternative to chemical control used in the study areas is the implementation of equalization cuts in pasture management. In this method, grass residues left by animals after grazing are cut. This technique would help control acaricide infestation by creating open, unprotected spaces in the paddocks. This alteration of the ecological niches allows sunlight to enter directly, which increases mortality in both adult ticks and larvae due to the effect of the alteration of the electrolyte balance and the evapotranspiration gradient [63, 64, 65]. Additionally, this technique helps to expose larvae to biological controllers in the soil [66].

The MCA helped to better understand the interaction between management variables, acaricide resistance, and tick infestation; so, as mentioned earlier, the application of different tick control strategies relies on farmers' uptake, knowledge, and perception of their effects. This kind of information is crucial for improving tick control management, particularly those practices that mitigate acaricide resistance and ensure long-term solutions that help to sustain the efficacy of tick control measures [30]. The use of alternative control practices was found to be related to farmers' knowledge about ticks, with those with a better level of knowledge seeking alternatives to chemical control. Although most farmers practicing alternative control methods belonged to Group 1, some were also found in Groups 2 and 3, where acaricide resistance and high tick infestation exist. This underscores that effective and successful control requires a combination of tools and strategy [30]. The use of alternative control must be accompanied by proper acaricide management. In addition, regardless of the treatment method, producers must be aware of and correctly apply control methods to obtain maximum benefits [67]. This can be achieved through farmer education since many farmers only use chemical control because they are unaware of other control methods [30, 67].

In this study, it was observed that the management of acaricides and the management of grazing systems had an impact on the presence or absence of high tick burdens on the animals. On the one hand, the high level of infestation was related to the use of extensive grazing and extreme frequencies of spray baths (very frequent or very infrequent treatments). We associate this with adequate tick control in animals, which requires the right combination of strategies [27–29]. Using an extensive grazing method causes animals to spend more time in contact with ticks, which contributes to increased infestation levels. As an effect of this, farmers, in their eagerness to combat high tick burdens, reduce the time between treatments (less than 15 days) [51, 68]. Although it could be seen that this was the main mode of action, there were also farmers that, despite having a high tick burden, applied acaricide baths infrequently (more than 5 weeks), which indicates that these farmers did not give importance to the presence of high tick loads, nor did they look for ways to control them.

On the other hand, it was determined that for the farms that managed a more intensive grazing system, the level of infestation was lower. Although this has already been reported in other studies [2, 69, 70], it is emphasized that this form of control must be accompanied by acaricide applications to reduce tick load from 77% to 89% [71]. Although this group of farmers used acaricide baths every 3 or 4 weeks, which is recommended to break the life cycle of these parasites [2], work should be done to educate farmers to treat only affected animals, since most of the farmers surveyed applied acaricide treatments to all animals, regardless of whether they required it or not.

Similarly, it was observed that farmers who tend to mix different acaricides also opt for other more extreme practices, including excessive and overdosage of acaricides (generally organophosphates) and the use of other irritant substances such as engine oil. These substances, in addition to irritating and causing skin lesions in animals, are toxic to livestock and humans if applied without adequate biosecurity measures [51, 72]. In addition, using engine oil is a risky practice that can cause food poisoning if it contaminates bovine products, which can affect human health long-term, even if consumed at very low doses [73, 74]. Pajurek et al. [74] presented a case of contamination of products of animal origin (eggs) with high levels of toxic substances (polychlorinated dibenzo-p-dioxin, dibenzofurans (PCDD/Fs)) due to the leakage of engine oil into the soil of the paddock where the animals were found.

### 4.2. Farmers' Knowledge

According to previous studies conducted as part of the project [7], it was determined that most of the respondents had at least 20 years of experience in cattle raising, so it is not surprising that the majority (93%) of the respondents were able to recognize the tick species found in the areas and perceived that its presence affected the farm's economy. However, not all farmers know the correct application of acaricides, the presence of larvae in the paddocks, and the predisposition to acaricide infestation in certain cattle breeds. This practical knowledge of ticks contrasted sharply with the lack of knowledge about TBDs and acaricide control regarding the correct dosage and rotation of acaricides. The lack of knowledge on these issues was already reported in studies by [75, 76], which further mentioned the existence of a directly proportional relationship between knowledge on TBDs and tick control with increasing levels of education and training courses for farmers. This makes sense since, in this study, most respondents have a basic or secondary level of education [7]. Although training is given at small rates, it is focused mainly on reproductive issues or good milking practices.

Given that farmers have limited access to formal education on ticks and TDB (training and university education), we can conclude that their knowledge is based on practical knowledge acquired through their farming experience and knowledge transmitted from generation to generation by their parents. Despite being informal, this knowledge can still be used to design and implement specific educational and training programs. These programs can help bridge the educational gap by incorporating traditional wisdom and introducing contemporary techniques to improve livestock management practices. This will empower livestock keepers with scientific knowledge and enhance their traditional techniques [77].

It is important to note that education programs should involve all decision-makers and stakeholders in acaricide control. Although the farmer–owner has the final decision on acaricide management, this decision may be influenced by suggestions or advice from livestock workers or neighboring farmers. Public veterinarians, while present in certain rural areas, usually provide technical advice on production and reproduction and leave sanitary control, including acaricide management, to private veterinarians, who may only be available to some farmers. Decision-making is also influenced by veterinary drug sellers, which in the case of large farms are veterinarians from pharmaceutical companies that offer their products from farm to farm; on the other hand, in the case of small and medium producers, they accept advice from store sellers, who in most cases have no veterinary training.

Training stakeholders, (1) farmers, (2) public and private veterinarians, and (3) commercial representatives of veterinary products, is crucial in acaricide control. The education of farmers, including both employees and owners, should be done through practical illustrated manuals or education programs. Studies have revealed that education and training programs designed with more interactive, communicative, and participatory approaches significantly impact the assimilation of information and the effective implementation of acaricidal control strategies [76, 78, 79]. It is crucial to train public and private veterinarians in tick control, as their involvement in this field is limited. The creation of technical manuals and training will help private veterinarians, public veterinarians, and veterinary drug sellers provide accurate technical advice and transmit their solid and updated knowledge to farmers.

In addition, involving authorities in socioeconomic and political reforms is crucial for successful long-term tick population management [17, 78]. The regulation of acaricide sales by trained personnel, establishment of acaricide resistance diagnosis laboratories, and implementation of socioeconomic and political reforms all play significant roles in tick management. To stimulate the adoption of new policies, the authorities must understand that although farmers are the main social group affected both by the economic losses caused by ticks and by being constantly exposed to acaricides (occupational exposure), the misuse of these chemicals exposes the general population if they consume food or drinking water contaminated with acaricide residues [80, 81].

### 4.3. Perceptions of Cattle Tick Seasonality

The perceptions of livestock farmers on seasonality and tick infestation in animals differed in the study areas despite both areas being humid subtropical zones. On the one hand, rainfall in the Quijos River valley is constant throughout the year, but there is a dry season with less intense precipitation from July to September [82, 83]. On the other hand, the Northwestern Pichincha has a more defined dry season, characterized by little or no precipitation between June and November [84]. It is associated that ranchers in the Quijos River valley area perceive tick infestation to be relatively constant throughout the year, as there is no marked difference between the two seasons. However, in the Northwest of Pichincha area, having a more defined dry season, there is a difference in the perception of a greater infestation by ticks in the months of June to November, months corresponding to the dry season. Farmers' perception of a higher prevalence of ticks during the months corresponding to the summer season is consistent with other studies where increased tick infestation in summer is associated with elevated temperature and humidity that stimulate tick development, survival, and spread [83, 85, 86, 87]. This perception of a higher level of infestation in the dry season may also be associated with the fact that in season, as the availability and quality of pasture decreases, animals spend more time in the paddocks, which exposes them for a longer time to ticks [88]. Moreover, at this time, as a result of the lack of paddocks, farmers in the areas move the animals to external paddocks, which increases the risk of infestation [7]. In addition, animals with low nutrition are more attacked by ticks [89].

### 4.4. Effect of Tick Infestation

According to the farmers' reports, the diseases affecting these tropical areas were observed. While TBDs were not the most prevalent disease in the study areas, they were the deadliest cause. Myiasis due to *D. hominis* was also reported by several farmers (52%), which can be related to the presence of ticks and poor acaricide control practices. The lesions caused by ticks or bad control practices (overdose, motor oil) allow the entry of bacteria, fungi, and parasites [90]. In addition, it was observed that due to the improper use of acaricides, there were reports of animals poisoned by acaricides, which died in most cases.

All farmers who participated in the participatory meetings reported that tick infestation negatively affected animal health and production. It was determined that decreased milk production, weight loss, and the presence of TBDs were the most frequent effects cited by farmers. Very few farmers mentioned exposure to acaricides and environmental pollution as potential effects, which is consistent with the low number of farmers with a good level of knowledge. By contrast, the nonuse of correct individual protective equipment and how farmers dispose of acaricide bottles are strong environmental concerns. When weighing the economic losses caused by ticks in three aspects: milk drop production, loss of weight, and skin damage. It was observed that the greatest weight in the proportional piling fell on the economic losses caused by the decrease in milk, which we associate with the fact that the study areas are dairy areas, and their main income is the sale of milk. Although most of the participants talked about the skin lesions caused by the presence of ticks or larvae of *D. hominis*, at the time of weighing this aspect in the economic losses, it was the one that received the least weight in the proportional piling. This indicates that although it is a visual problem that bothers the farmers, at the moment of selling the animals, the good or bad condition of the skin does not interfere with the remuneration received for their sale.

## 5. Conclusions

The application of tick control strategies strongly relies on farmers' uptake, knowledge, and perceptions of the control effects [30]. This study identified tick control practices used by dairy farms in subtropical areas and described common and uncommon measures used. In our study, farmers with good knowledge had lower infestation rates and acaricide resistance. They were also most active in the use of alternative methods. While cross-sectional data cannot establish a causal relationship, our results strongly indicate that a good understanding of tick biology and well-informed use of tick control is key for tick management. The right combination of acaricidal control strategies can increase the efficacy of control methods, making them more cost-effective and ecologically sustainable [91]. Based on this and previous studies, the importance of working on three points is highlighted: (1) the implementation of an integrated tick-control program that allows evaluating the effectiveness of the strategies and the farmer uptake; (2) the education of farmers through the creation of extension programs with interactive, communicative, and participatory approaches and (3) the creation of working groups with authorities, national, and international experts. These three points together will help to promote the creation of sanitary control policies. Understanding the complex interplay between farmers' characteristics, practices, and perceptions is vital to developing well-informed strategies. By exploring these dynamics, farmers can tailor interventions to meet the specific needs and challenges faced by livestock in the country. This approach enables the development of more effective control measures that align with the reality of livestock management practices in Ecuador. The implementation of customized interventions not only mitigates economic losses associated with tick infestations but also enhances livestock health and productivity, benefiting both producers and consumers. This will help to control the advance of resistant tick populations, a global problem in which FAO [92] works in multidisciplinary and multi-sectoral programs to plan and collaborate in the sustainable management of cattle ticks. This kind of information is also crucial for improving tick control management in Ecuador, particularly to try to implement practices that mitigate acaricide resistance and ensure long-term solutions that help to sustain the efficacy of tick control treatments.

## Figures and Tables

**Table 1:** Active components used to control ticks on cattle in Ecuador.

Acaricide (approximate date introduced ^*∗*^)	Active compounds ^*∗∗*^	Site of action	Mode of action	Reference
Organophosphates (1950)	Ethion, chlorpyrifos, coumaphos, dichlorvos, and trichlorfon	Nervous system	Acetylcholine esterase inhibitors	[8]

Amidines (1970)	Amitraz	Nervous system	Octopamine agonists	[9, 10]

Synthetic pyrethroids (1970)	Alpha-cypermethrin, cypermethrin, deltamethrin, flumethrin and permethrin	Nervous system	Sodium channel modulators	[11, 12, 13]

Macrocyclic lactones (1981)	Doramectin, ivermectin, and eprinomectin	Nervous system	Glutamate-controlled chloride channel activator	[14]

Phenylpyrazoles (1990)	Fipronil	Nervous system	Blocking GABA mediated chloride channels	[15]

Benzoylphenyl ureas (1994)	Fluazuron	Exoskeleton	Inhibiting chitin incorporation into the tick's cuticle	[16]

^*∗*^Approximate date of Introduction of acaricides into the global market;  ^*∗∗*^Active ingredients have been registered until 2023 by the Phytosanitary and Zoo sanitary Regulation and Control Agency (Ecuador); GABA, gamma-aminobutyric acid.

**Table 2:** Control and perception variables used in the study.

Topic	Variable	Source of information
Tick control practices	Percentage efficacy of acaricides	Cross-sectional survey
	Chemical control: frequency, acaricide dynamics used dosage, route of administration, who prepares the acaricide solution, and treated animals	
	Alternative control practices used	
	Type of grazing implemented	

Farmers' perception	Effect of tick infestation	Cross-sectional survey and participatory meeting
	Seasonality of tick infestation	

Farmers' knowledge	Biology of ticks Breed predisposition Tick-borne diseases Knowledge of economic losses caused by ticks Correct acaricide treatment	Cross-sectional survey

Direct and indirect economic losses	Price (USD) per milligram or gram of acaricide (active component)	Agro warehouses interview and cross-sectional survey
	Cost (USD) treatment by animal	
	Economic losses in milk, beef, and hide	Participatory meeting

Tick infestation	Level of tick infestation at the farm level	Paucar et al. [7]

Acaricide resistance	Presence or absence of resistance in three acaricides	Larval package test

**Table 3:** Variables used for the multicomponent analysis (*N* = 126 farmers considered).

Variable	Categories	Farms	Codification
Presence of a high level of tick infestation	No	70	low infestation
	Yes	56	high infestation

Amitraz resistance	No	61	am res no
	Yes	65	am res yes

Ivermectin resistance	No	74	iv res no
	Yes	52	iv res yes

Alpha-cypermethrin resistance	No	58	cy res no
	Yes	68	cy res yes

Multiresistance: amitraz and ivermectin	No	94	am iv res no
	Yes	32	am iv res yes

Multiresistance: amitraz and alpha-cypermethrin	No	83	am cy res no
	Yes	43	am cy res yes

Multiresistance: alpha-cypermethrin and ivermectin	No	91	cy iv res no
	Yes	35	cy iv res yes

Multiresistance: amitraz, alpha-cypermethrin, and ivermectin	No	101	X3res no
	Yes	25	X3res yes

Who preparated the acaricide treatment	Employed	27	Employed
	Owner	99	Owner

Mixture of different acaricides	No	95	acaricide mix no
	Yes	31	acaricide mix yes

Add additives	No	114	additives no
	Yes	12	additives yes

Acaricide application with a wipe cloth	No	113	appl wipe no
	Yes	13	appl wipe yes

Use of organophosphates	No	47	org use no
	Yes	79	org use yes

Alternative acaricide control	No	76	alt no
	Yes	50	alt yes

Manual removal of ticks	No	84	m removal no
	Yes	42	m removal yes

Frequency of bath sprays	1 or 2 weeks	55	bath 1–2
	3 or 4 weeks	42	bath 3–4
	5 weeks or more	21	bath >5

Reported efficacy: bath spray	High-efficacy bath	23	high eff bath
	Low-efficacy bath	29	low eff bath
	Medium-efficacy bath	60	med eff bath

Reported efficacy: injection	High-efficacy bath	50	high eff injection
	Low-efficacy bath	22	low eff injection
	Medium-efficacy bath	29	med eff injection

Tick-borne diseases report	No	67	TBDs no
	Yes	59	TBDs yes

Knowledge level	Fair knowledge ^*∗*^	91	fair knowledge
	Good knowledge	35	good knowledge

Grazing system	Extensive ^*∗∗*^	30	ext graz
	Intensive ^*∗∗*^	96	int graz

^*∗*^Due to the small amount of data from farmers with poor knowledge, they were grouped into the group with fair knowledge;  ^*∗∗*^Intensive: grazing in small area enclosed paddocks, where the animals remain for short periods of occupation (1 day); Extensive: grazing in large areas paddocks where cattle remain for longer periods (more than 1 day).

**Table 4:** Chemical control of ticks: practices, perceived efficacy by farmers (*N* = 136) and cost of treatment.

Acaricide	*N*	Reported efficacy (%)	Doses (ml/g)			Cost treatment by animal (UDS)		
			Prescribed dose	Used dose	*p*-Value	Prescribed dose	Used dose	*p*-Value
(a) Bath spray (ml or gr used by *L*)								
Amides	97	59	1.00	1.28 (1.17–1.40)	<0.01^*∗∗*^	0.37	0.47 (0.43–0.51)	<0.01^*∗∗*^
Pyrethroid	52	59	1.00	1.35 (1.10–1.59)	0.01^*∗∗*^	0.36	0.49 (0.40–0.57)	0.01^*∗∗*^
Pyrethroid + organophosphate	41	67	1.00	1.30 (1.13–1.46)	<0.01^*∗∗*^	0.37	0.48 (0.42–0.54)	<0.01^*∗∗*^
Pyrethroid + organophosphate + phenylpyrazolone	32	67	1.00	1.19 (1.00–1.38)	0.05^*∗*^	0.36	0.43 (0.36–0.50)	0.05^*∗*^
Organophosphate (liquid)	28	71	1.50	1.45 (1.00–1.90)	0.82	0.26	0.25 (0.17–0.32)	0.75
Organophosphates (powder)	33	67	1.50	0.98 (0.85–1.10)	<0.01^*∗∗*^	0.55	0.36 (0.32–0.41)	<0.01^*∗∗*^
(b) Injection (ml used by 50 kg weight)								
Macrocyclic lactone 1%	89	70	1.00	1.05 (0.94–1.17)	0.36	1.17	1.10 (1.02–1.18)	0.09
Macrocyclic lactone >1%	32	88	1.00	1.10 (0.94–1.26)	0.20	1.60	1.76 (1.51–2.00)	0.20
(c) Pour-on (ml used by 10 kg weight)								
Benzoylphenyl urea	19	83	1.00	0.86 (0.77–0.95)	<0.01^*∗∗*^	3.62	2.96 (2.48–3.44)	0.01^*∗∗*^
Benzoylphenyl urea + phenylpyrazolone	24	91	1.00	0.69 (0.55–0.84)	<0.01^*∗∗*^	2.14	1.45 (1.11–1.80)	<0.01^*∗∗*^

The cost of acaricide treatment was calculated for a 400 kg adult animal.  ^*∗*^Statistically significant (according to *t*-test, *p*  < 0.05);  ^*∗∗*^Statistically significant (according to *t*-test, *p*  < 0.01). Acaricides with phenylpyrazolone or benzoylphenyl urea + macrocyclic lactone as active ingredients were discarded from this table due to insufficient data.

**Table 5:** Characterization of groups formed in hierarchical classification.

Variable	Group 1		Group 2		Group 3		Total farms
	*N*	%	*N*	%	*N*	%	
Presence of high level of tick infestation	15	39	17	57	9	50	41
Without resistance	11	29	7	23	0	0	18
Mono acaricide resistance	16	42	11	37	0	0	27
Amitraz resistance	13	34	13	43	18	100	44
Ivermectin resistance	10	26	7	23	18	100	35
Alpha-cypermethrin resistance	15	39	17	57	18	100	50
Multiresistance to two acaricides	11	37	14	47	18	100	43
Multiresistance: amitraz and ivermectin resistance	3	8	1	3	18	100	22
Multiresistance: amitraz and alpha-cypermethrin resistance	5	13	9	30	18	100	32
Multiresistance: alpha-cypermethrin and ivermectin resistance	3	8	4	13	18	100	25
Multiresistance to three acaricides	0	0	0	0	18	100	18
Frequency of acaricide bath spray: 1 or 2 weeks	15	39	17	57	9	50	41
Frequency of acaricide bath spray: 3 or 4 weeks.	18	47	8	27	6	33	32
Frequency of acaricide bath spray: 5 or more weeks	5	13	5	17	3	17	13
Low efficacy in acaricide treatments applied in spray baths	3	8	12	40	6	33	21
Medium efficacy in acaricide treatments applied in spray baths	24	63	13	43	10	56	47
High efficacy in acaricide treatments applied in spray baths	11	29	5	17	2	11	18
Low efficacy in acaricide treatments applied in injection	5	13	10	33	4	22	19
Medium efficacy in acaricide treatments applied in injection	6	16	14	47	5	28	25
High efficacy in acaricide treatments applied in injection	27	71	6	20	9	50	42
Good level of knowledge	12	32	6	20	7	39	25
Application with wipe cloth	0	0	4	13	3	17	7
Use of organofosforados	18	47	28	93	14	78	60
Mixture of different acaricides	5	13	16	53	2	11	23
Intensive grazing	32	84	16	53	10	56	58
Add additives	8	21	1	3	1	6	10
Alternative acaricide control ^*∗*^	32	84	10	33	13	72	55
Tick-borne diseases report	13	34	18	60	10	56	41
Owner was responsible for preparing and applying the acaricide treatments.	34	89	27	90	11	61	72

^*∗*^Alternative acaricidal control includes equalization cuts, entomopathogenic fungal dips, herbal extracts, sulfur, or manual tick removal. Mono resistance refers to farms where there is resistance to a single acaricide.

**Table 6:** Summary scoring matrix of economic losses in milk, weight, and hide on dairy farms in tropical areas.

Study area	*N*	High infestation level	Kendall's coefficient	Economic losses					
				Milk		Weight		Hide	
Quijos River valley	13	No	0.53^*∗∗*^	••••	*p*-Value 1.00	**••••**	*p*-Value 0.28	**••••**	*p*-Value 0.42
				••				•	
				6 (4−8)		4 (2–5)		5 (3–7)	
	14	Yes	0.16	**••••**		•••		••••	
				**••••**					
				8 (6–9)		3 (2–5)		4 (2–6)	

Northwest of Pichincha	8	No	0.84^*∗∗*^	**••••**	*p*-Value 0.04^*∗*^	**••••**	*p*-Value 0.76	••	*p*-Value 0.06
				**••••**		••			
				8 (7–9)		6 (4–7)		2 (0–3)	
	5	Yes	0.74^*∗∗*^	••••		••••			
				••••		•••			
				8 (6–9)		7 (5–9)		0 (0–2)	

*N* = number of informant groups. *W* = Kendall's coefficient of concordance ( ^*∗*^*p*  < 0.05;  ^*∗∗*^*p*  < 0.01). *W* values vary from 0 to 1.0; the higher the value, the higher the level of agreement between the informant groups. The black dots (**•**) represent the median scores (number of marble balls) used during the matrix scoring; 95% confidence limits are shown in parentheses.  ^*∗*^Statistically significant (according to Mann–Whitney test, *p*  < 0.05).

## Data Availability

The data that support the findings of this study are available from the corresponding author upon request.

## Supplementary Materials

Supplementary 1 Figure S1: graphical abstract of the study.

Supplementary 2 Figure S2: image used to classify farms into no, low, medium, and high tick infestation.

## References

[1] Reck J., Klafke G. M., Webster A. (2014). First report of fluazuron resistance in *Rhipicephalus microplus*: a field tick population resistant to six classes of acaricides. *Veterinary Parasitology*.

[2] Muhammad G., Naureen A., Firyal S., Saqib M. (2008). Tick control strategies in dairy production medicine. *Pakistan Veterinary Journal*.

[3] Muyobela J., Nkunika P. O. Y., Mwase E. T. (2015). Resistance status of ticks (Acari; Ixodidae) to amitraz and cypermethrin acaricides in Isoka District, Zambia. *Tropical Animal Health and Production*.

[4] Pérez X. (2016). Resistance to alpha-cipermethrin, ivermectin and amitraz in ticks *Rhipicephalus microplus* (Canestrini, 1887) collected in four localities. http://www.dspace.uce.edu.ec/handle/25000/10254.

[5] Orozco G. (2018). Spatial distribution of ticks that affect Ecuadorian livestock in the three regions, using the equinoctial line as a reference. https://www.dspace.uce.edu.ec/handle/25000/32280.

[6] Maya-Delgado A., Madder M., Benítez-Ortíz W., Saegerman C., Berkvens D., Ron-Garrido L. (2020). Molecular screening of cattle ticks, tick-borne pathogens and amitraz resistance in ticks of Santo Domingo de los Tsáchilas province in Ecuador. *Ticks and Tick-Borne Diseases*.

[7] Paucar V., Pérez-Otáñez X., Rodríguez-Hidalgo R. (2022). The associated decision and management factors on cattle tick level of infestation in two tropical areas of Ecuador. *Pathogens*.

[8] Smith G. J. (1987). *Pesticide Use and Toxicology in Relation to Wildlife: Organophosphorus and Carbamate Compounds*.

[9] Curtis R. J. (1985). Amitraz in the control of non-ixodide ectoparasites of livestock. *Veterinary Parasitology*.

[10] Hollingworth R. M. (1976). Chemistry, biological activity, and uses of formamidine pesticides. *Environmental Health Perspectives*.

[11] Narahashi T. (1971). Mode of action of pyrethroids. *Bulletin of the World Health Organization*.

[12] Pang G.-F. (2018). Introduction. *Analytical Methods for Food Safety by Mass Spectrometry*.

[13] Davies T. G. E., Field L. M., Usherwood P. N. R., Williamson M. S. (2007). DDT, pyrethrins, pyrethroids and insect sodium channels. *IUBMB Life*.

[14] Vercruysse J. (2002). *Macrocyclic Lactones in Antiparasitic Therapy*.

[15] Beasley V. R. (2020). Direct and indirect effects of environmental contaminants on amphibians. *Reference Module in Earth Systems and Environmental Sciences*.

[16] Junquera P., Hosking B., Gameiro M., Macdonald A. (2019). Benzoylphenyl ureas as veterinary antiparasitics. An overview and outlook with emphasis on efficacy, usage and resistance. *Parasite*.

[17] FAO (2004). *Guidelines Resistance Management and Integrated Parasite Control in Rumiants*.

[18] Agrocalidad (2023). Report of registered veterinary products.

[19] De Oliveira P. R., Calligaris I. B., Roma G. C., Bechara G. H., Pizano M. A., Camargo Mathias M. I. (2012). Potential of the insect growth regulator, fluazuron, in the control of *Rhipicephalus sanguineus* nymphs (Latreille, 1806) (Acari: Ixodidae): determination of the LD95 and LD50. *Experimental Parasitology*.

[20] Calligaris I. B., De Oliveira P. R., Roma G. C., Bechara G. H., Camargo-Mathias M. I. (2013). Action of the insect growth regulator fluazuron, the active ingredient of the acaricide Acatak®, in *Rhipicephalus sanguineus* nymphs (Latreille, 1806) (Acari: Ixodidae). *Microscopy Research and Technique*.

[21] Tinoco T. (2022). Evaluation of the effectiveness of the GAVAC immunogen and the rational use of acaricides as an alternative for an integrated tick control program. http://www.dspace.uce.edu.ec/handle/25000/27697.

[22] Rodríguez-Hidalgo R., Pérez-Otáñez X., Garcés-Carrera S. (2017). The current status of resistance to alpha-cypermethrin, ivermectin, and amitraz of the cattle tick (*Rhipicephalus microplus*) in Ecuador. *PLOS ONE*.

[23] Paucar-Quishpe V., Pérez-Otáñez X., Rodríguez-Hidalgo R. (2023). An economic evaluation of cattle tick acaricide-resistances and the financial losses in subtropical dairy farms of Ecuador: a farm system approach. *PLOS ONE*.

[24] Akhtar S. (2017). Pesticides residue in milk and milk products: mini review. *Pakistan Journal of Analytical & Environmental Chemistry*.

[25] Laffont C. M., Alvinerie M., Bousquet-Mélou A., Toutain P.-L. (2001). Licking behaviour and environmental contamination arising from pour-on ivermectin for cattle. *International Journal for Parasitology*.

[26] Rehman H., Munir M., Ashraf K. (2022). Heavy metals, pesticide, plasticizers contamination and risk analysis of drinking water quality in the newly developed housing societies of Gujranwala, Pakistan. *Water*.

[27] FAO (2004). *Resistance Management and Integrated Parasite Control in Rumiants*.

[28] Rodriguez-Vivas R. I., Jonsson N. N., Bhushan C. (2018). Strategies for the control of *Rhipicephalus microplus* ticks in a world of conventional acaricide and macrocyclic lactone resistance. *Parasitology Research*.

[29] Humblet M.-F., Losson B., Saegerman C. (2020). Integrated management of blood-feeding arthropods in veterinary teaching facilities-part 1: overview of haematophagous arthropods of interest in north-western Europe. *Revue Scientifique et Technique de l’OIE*.

[30] Jack C., Hotchkiss E., Sargison N. D., Toma L., Milne C., Bartley D. J. (2022). Determining the influence of socio-psychological factors on the adoption of individual ‘best practice’ parasite control behaviours from Scottish sheep farmers. *Preventive Veterinary Medicine*.

[31] Van Huis A. (1981). Integrated pest management in the small farmer’s maize crop in Nicaragua. *Wageningen*.

[32] Haro R. (2003). Report on animal genetic resources Ecuador. http://www.fao.org/ag/againfo/programmes/en/genetics/documents/Interlaken/countryreports/Ecuador.pdf.

[33] Muñoz E. C. (2023). Grazing management strategies adapted to dairy cattle on pasture in the ecuadorian sierra. https://hdl.handle.net/2268/304599.

[34] Lupo C., Wilmart O., Van Huffel X., Dal Pozzo F., Saegerman C. (2016). Stakeholders’ perceptions, attitudes and practices towards risk prevention in the food chain. *Food Control*.

[35] Catley A., Alders R. G., Wood J. L. N. (2012). Participatory epidemiology: approaches, methods, experiences. *The Veterinary Journal*.

[36] Pesquera C., Portillo A., Palomar A. M., Oteo J. A. (2015). Investigation of tick-borne bacteria (*Rickettsia* spp., *Anaplasma* spp., *Ehrlichia* spp. and *Borrelia* spp.) in ticks collected from Andean tapirs, cattle and vegetation from a protected area in Ecuador. *Parasites & Vectors*.

[37] Guglielmone A. A., Robbins R. G. (2018). *Hard Ticks (Acari: Ixodida: Ixodidae) Parasitizing Humans*.

[38] Enríquez S., Guerrero R., Arrivillaga-Henríquez J. (2020). New records of ticks of genus *Amblyomma* Koch, 1844 (Acari: Ixodidae) for Ecuador. *Acta Parasitologica*.

[39] FAO (2011). Participatory epidemiology-methods for action and data collection aimed at epidemiological intelligence.

[40] R Core Team (2021). R: a language and environment for statistical computing. https://www.r-project.org/.

[41] Siegel S. (1957). Nonparametric statistics. *The American Statistician*.

[42] Gower J., Lubbe S., Roux N.le (2010). Multiple correspondence analysis. *Understanding Biplots*.

[43] Lê S., Josse J., Husson F. (2008). FactoMineR: an *R* package for multivariate analysis. *Journal of Statistical Software*.

[44] Kassambara A., Mundt F. (2020). Factoextra: extract and visualize the results of multivariate data analyses.

[45] Coffin J. L., Monje F., Asiimwe-Karimu G., Amuguni H. J., Odoch T. (2015). A one health, participatory epidemiology assessment of anthrax (*Bacillus anthracis*) management in Western Uganda. *Social Science & Medicine*.

[46] Mugisha A., McLeod A., Percy R., Kyewalabye E. (2005). Strategies, effectiveness and rationale of vector-borne disease control in the pastoralist system of south-western Uganda. *Tropical Animal Health and Production*.

[47] Mugabi K. N., Mugisha A., Ocaido M. (2010). Socio-economic factors influencing the use of acaricides on livestock: a case study of the pastoralist communities of Nakasongola District, Central Uganda. *Tropical Animal Health and Production*.

[48] Vudriko P., Okwee-Acai J., Byaruhanga J. (2018). Chemical tick control practices in southwestern and northwestern Uganda. *Ticks and Tick-Borne Diseases*.

[49] Obaid M. K., Islam N., Alouffi A. (2022). Acaricides resistance in ticks: selection, diagnosis, mechanisms, and mitigation. *Frontiers in Cellular and Infection Microbiology*.

[50] Cuore U., Trelles A., Sanchis J., Gayo V., Solari M. A. (2007). First diagnosis of resistance to Fipronil in the common cattle tick *Boophilus microplus*. *Veterinary (Montevideo)*.

[51] Vudriko P., Okwee-Acai J., Tayebwa D. S. (2016). Emergence of multi-acaricide resistant *Rhipicephalus* ticks and its implication on chemical tick control in Uganda. *Parasites & Vectors*.

[52] Adakal H., Stachurski F., Chevillon C. (2013). Tick control practices in Burkina Faso and acaricide resistance survey in *Rhipicephalus* (*Boophilus*) *geigyi* (Acari: Ixodidae). *Experimental and Applied Acarology*.

[53] Tapias V. M. (2011). Resistance of *Rhipicephalus* (*Boophilus*) *microplus* (Canestrini, 1888) to acaricides in cattle in dairy farms, Cercado province, Beni, Bolivia. *Scientific Journal Agrociencias*.

[54] WingChing Jones R. (2015). Manual removal of *Rhipicephalus* (*Boophilus*) *microplus* ticks from cattle as a control strategy. *Tropical Animal Nutrition*.

[55] Herrera L., Romero A. (2022). Control of tick nymphs (*Rhipicephalus microplus*) in combination with *Beauveria* spp. with organic and chemical molecules. http://repositorio.espe.edu.ec/handle/21000/32381.

[56] Hidalgo D., Tello C. (2022). Manual for the production of entomopathogenic fungi and quality analysis of bioformulates. *National Institute of Agricultural Research*.

[57] Masapanta L. (2019). Efficacy of *Metarhizium* spp. in the control of adult ticks under in vitro conditions. https://repositorio.uea.edu.ec/handle/123456789/367.

[58] Valle S., Caicedo W., Masapanta L. (2020). Efficacy of a native isolation of *Metarhizium anisopliae* (TI6301) for the control of adult ticks (*Rhipicephalus microplus*) under in vitro conditions. *Livestock Research for Rural Development*.

[59] Costa L. M., Furlong J. (2011). Efficiency of sulphur in garlic extract and non-sulphur homeopathy in the control of the cattle tick *Rhipicephalus* (*Boophilus*) *microplus*. *Medical and Veterinary Entomology*.

[60] Giglioti R., Forim M. R., Oliveira H. N. (2011). In vitro acaricidal activity of neem (*Azadirachta indica*) seed extracts with known azadirachtin concentrations against *Rhipicephalus microplus*. *Veterinary Parasitology*.

[61] Egas J., Lopez S. (2023). Evaluation of the effect of elemental sulfur as a tick control alternative and its influence on milk production in cattle farms in the northwest of Pichincha. https://www.dspace.uce.edu.ec/handle/25000/32280.

[62] James D. G., Prischmann D., Sabelis M. W., Bruin J. (2010). The impact of sulfur on biological control of spider mites in Washington State vineyards and hop yards. *Trends in Acarology*.

[63] Hernández F. (2005). Integrated management in tick control. *Dual Purpose Livestock Manual*.

[64] Nielebeck C., Kim S. H., Pepe A. (2023). Climatic stress decreases tick survival but increases rate of host-seeking behavior. *Ecosphere*.

[65] Salazar B R., Barahona-Rosales R., Sánchez P M.-S. (2016). Tick loads in *Bos taurus* cattle grazing in two contrasting production systems. *Revista MVZ Córdoba*.

[66] Samish M., Rehacek J. (1999). Pathogens and predators of ticks and their potential in biological control. *Annual Review of Entomology*.

[67] George J. E. (2000). Present and future technologies for tick control. *Annals of the New York Academy of Sciences*.

[68] Miyama T., Byaruhanga J., Okamura I. (2020). Effect of chemical tick control practices on tick infestation and *Theileria parva* infection in an intensive dairy production region of Uganda. *Ticks and Tick-Borne Diseases*.

[69] Farley J., Filho A. L. S., Alvez J., Ribeiro de Freitas N. (2012). How valuing nature can transform agriculture. *Solutions*.

[70] Cruz-González G., Pinos-Rodríguez J. M., Alonso-Díaz M. Á. (2023). Rotational grazing modifies *Rhipicephalus microplus* infestation in cattle in the humid tropics. *Animals*.

[71] Abbas R. Z., Zaman M. A., Colwell D. D., Gilleard J., Iqbal Z. (2014). Acaricide resistance in cattle ticks and approaches to its management: the state of play. *Veterinary Parasitology*.

[72] Rajput Z. I., Hu S.-H., Chen W.-J., Arijo A. G., Xiao C.-W. (2006). Importance of ticks and their chemical and immunological control in livestock. *Journal of Zhejiang University SCIENCE B*.

[73] Saegerman C., Pussemier L., Huyghebaert A., Scippo M. L., Berkvens D. (2006). Contaminación de animales con sustancias químicas en las explotaciones. *Revue Scientifique et Technique de l’OIE*.

[74] Pajurek M., Mikolajczyk S., Warenik-Bany M. (2023). Engine oil from agricultural machinery as a source of PCDD/Fs and PCBs in free-range hens. *Environmental Science and Pollution Research*.

[75] Adehan S. B., Adakal H., Gbinwoua D. (2018). West African cattle farmers’ perception of tick-borne diseases. *EcoHealth*.

[76] Sungirai M., Moyo D. Z., De Clercq P., Madder M. (2016). Communal farmers’ perceptions of tick-borne diseases affecting cattle and investigation of tick control methods practiced in Zimbabwe. *Ticks and Tick-Borne Diseases*.

[77] Wanzala W., Zessin K. H., Kyule N. M., Baumann M. P. O., Mathias E., Hassanali A. (2005). Ethnoveterinary medicine: a critical review of its evolution, perception, understanding and the way forward. *Livestock Research for Rural Development*.

[78] Hu Z. (2020). What socio-economic and political factors lead to global pesticide dependence? A critical review from a social science perspective. *International Journal of Environmental Research and Public Health*.

[79] Stone G. D. (2016). Towards a general theory of agricultural knowledge production: environmental, social, and didactic learning. *Culture, Agriculture, Food and Environment*.

[80] Damalas C. A., Eleftherohorinos I. G. (2011). Pesticide exposure, safety issues, and risk assessment indicators. *International Journal of Environmental Research and Public Health*.

[81] Mutavi F., Heitkönig I., Wieland B., Aarts N., Van Paassen A. (2021). Tick treatment practices in the field: Access to, knowledge about, and on-farm use of acaricides in Laikipia, Kenya. *Ticks and Tick-Borne Diseases*.

[82] ECMWF (2021). Climate data. https://en.climate-data.org/Info/Sources/.

[83] Ullah N., Durrani A. Z., Avais M. (2018). A first report on prevalence of caprine theileriosis and its association with host biomarkers in Southern Khyber Pakhtunkhwa, Pakistan. *Small Ruminant Research*.

[84] Varela L. A., Ron S. R. (2018). Geography and climate of Ecuador.

[85] Katiyatiya C. L. F., Muchenje V., Mushunje A. (2014). Farmers’ perceptions and knowledge of cattle adaptation to heat stress and tick resistance in the Eastern Cape, South Africa. *Asian-Australasian Journal of Animal Sciences*.

[86] Velusamy R., Rani N., Ponnudurai G. (2014). Influence of season, age and breed on prevalence of haemoprotozoan diseases in cattle of Tamil Nadu, India. *Veterinary World*.

[87] Khan S. S., Ahmed H., Afzal M. S., Khan M. R., Birtles R. J., Oliver J. D. (2022). Epidemiology, distribution and identification of ticks on livestock in Pakistan. *International Journal of Environmental Research and Public Health*.

[88] Hernández-A F., Teel P. D., Corson M. S., Grant W. E. (2000). Simulation of rotational grazing to evaluate integrated pest management strategies for *Boophilus microplus* (Acari: Ixodidae) in Venezuela. *Veterinary Parasitology*.

[89] Tolleson D. R., Carstens G. E., Welsh T. H. (2012). Plane of nutrition by tick-burden interaction in cattle: effect on growth and metabolism. *Journal of Animal Science*.

[90] Namgyal J., Tenzin T., Checkley S. (2021). A knowledge, attitudes, and practices study on ticks and tick-borne diseases in cattle among farmers in a selected area of eastern Bhutan. *PLOS ONE*.

[91] Cameron M. M., Bell M., Howard A. F. V. (2013). Integrated vector management. *Biological and Environmental Control of Disease Vectors*.

[92] FAO (2023). Acaricide resistance community of practice page.

