# Supplementary figures and images for: Farmers' Adoption, Knowledge, and Perceptions of Tick Control Measures on Dairy Farms in Subtropical Areas of Continental Ecuador
Source: Transbound Emerg Dis. 2024 May 24;2024:5023240. doi: 10.1155/2024/5023240 (PMC12019928; doi:10.1155/2024/5023240)

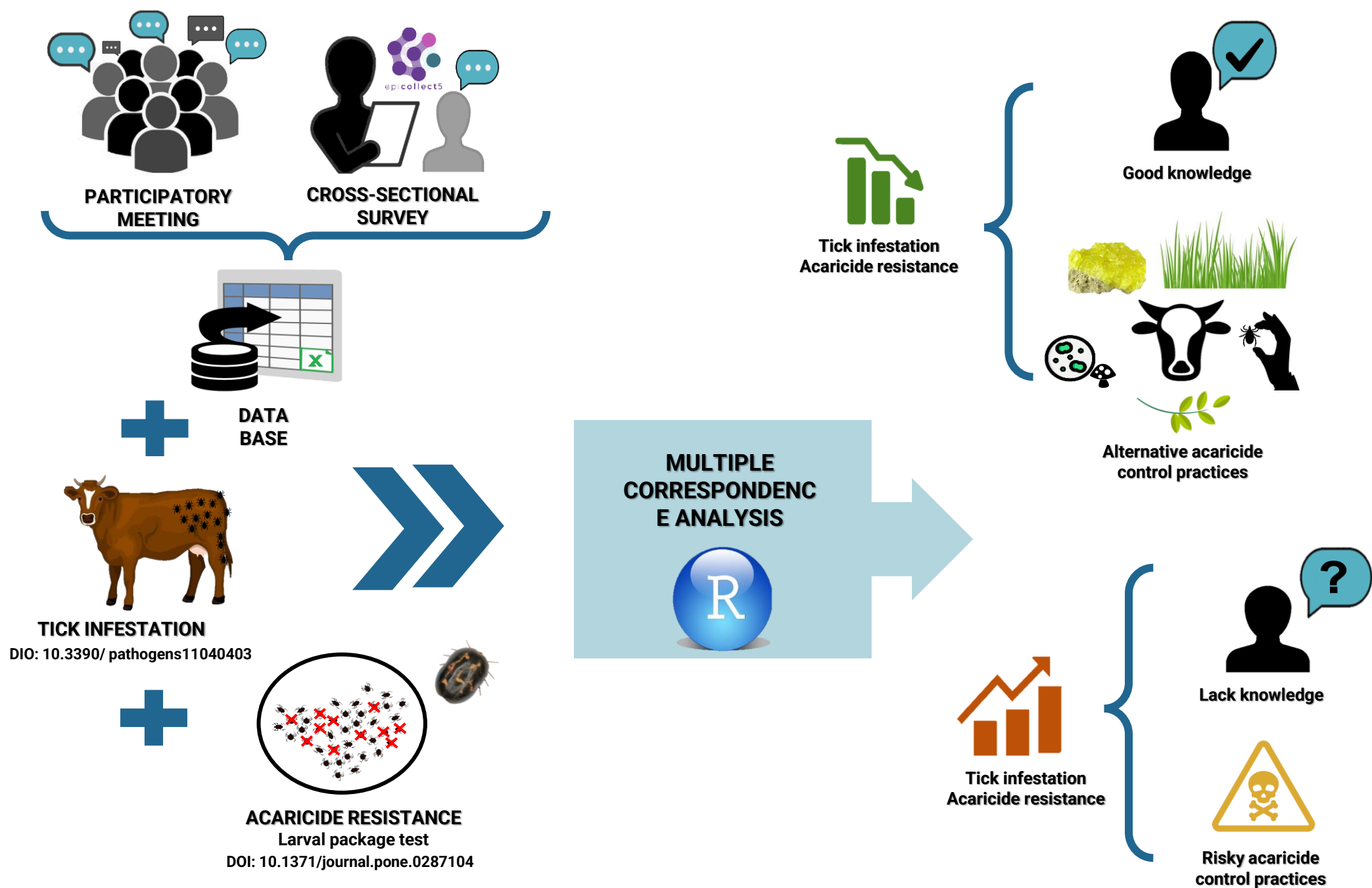

**Figure S1.** Graphical abstract of the study

Supplement: Supplementary 1 — Figure S1: graphical abstract of the study. [file 5023240.f1.pdf]
