# Supplementary material for: Farmers' Adoption, Knowledge, and Perceptions of Tick Control Measures on Dairy Farms in Subtropical Areas of Continental Ecuador
Source: Transbound Emerg Dis. 2024 May 24;2024:5023240. doi: 10.1155/2024/5023240 (PMC12019928; doi:10.1155/2024/5023240)
Supplement: Supplementary 2 — Figure S2: image used to classify farms into no, low, medium, and high tick infestation. [file 5023240.f2.pdf]

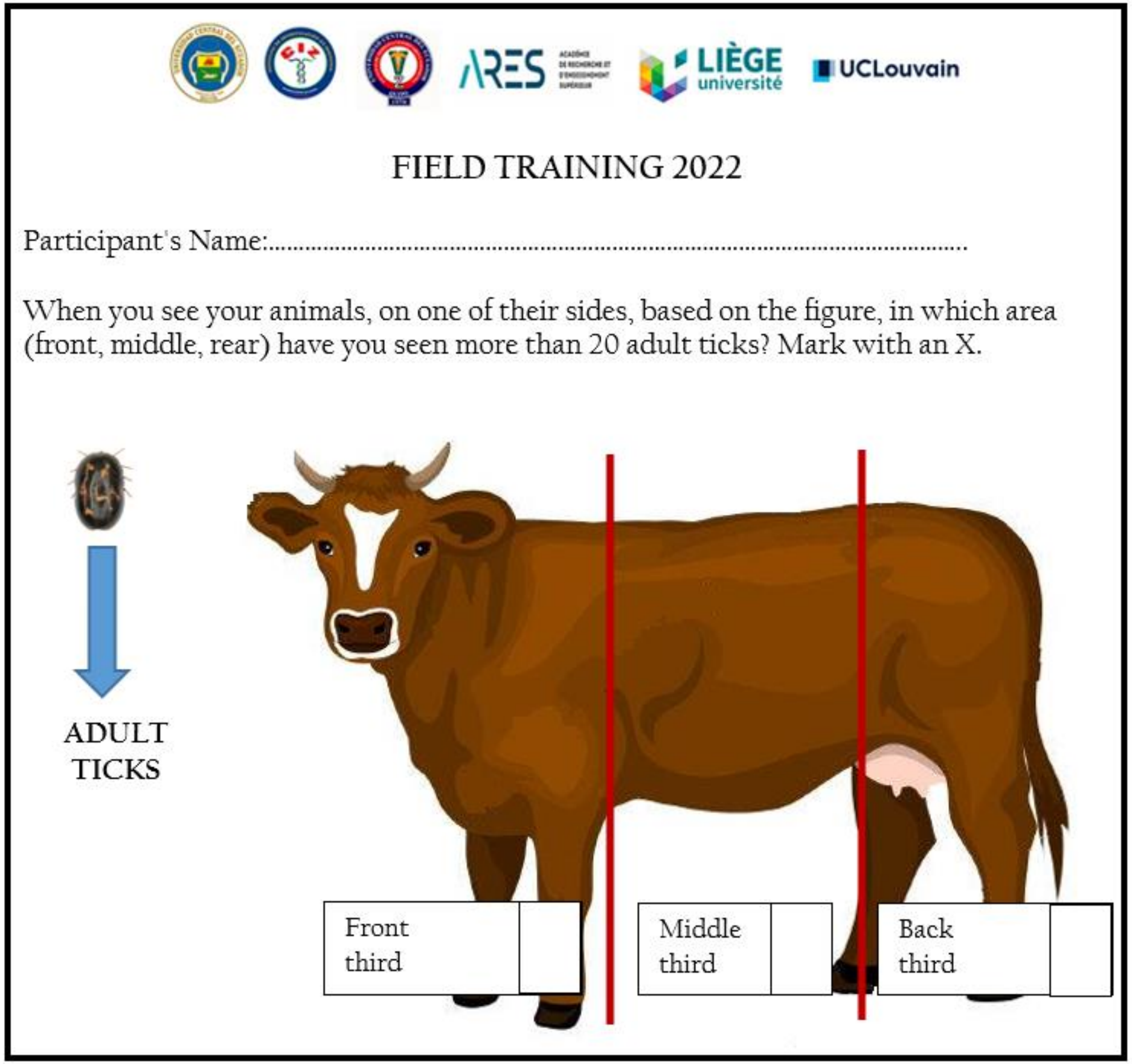

Figure S2. Image used to classify farms into no, low, medium, and high tick infestation
